# *In vivo* Assessment of the Impact of Molecular Weight on Constructs of ^68^Ga-DOTA-Manocept in a Syngeneic Mouse Tumor Model

**DOI:** 10.1007/s11307-023-01809-6

**Published:** 2023-03-07

**Authors:** Jennifer L. Bartels, Solana R. Fernandez, Jeffrey S. Arnold, Candace C. Parker, Volkan Tekin, Grace O’Malley, David A. Ralph, Suzanne E. Lapi

**Affiliations:** 1 Department of Radiology, The University of Alabama at Birmingham, Birmingham, AL 35294 USA; 2 Navidea Biopharmaceuticals, 4995 Bradenton Ave, Dublin, OH 43017 USA

**Keywords:** DOTA-tilmanocept, Syngeneic mouse tumor model, Molecular weight comparison, CD206 PET imaging

## Abstract

**Purpose:**

Manocept™ constructs are mannosylated amine dextrans (MADs) that bind with high affinity to the mannose receptor, CD206. Tumor-associated macrophages (TAMs) are the most numerous immune cells in the tumor microenvironment and a recognized target for tumor imaging and cancer immunotherapies. Most TAMs express CD206, suggesting utility of MADs to deliver imaging moieties or therapeutics to TAMs. The liver Kupffer cells also express CD206, making them an off-target localization site when targeting CD206 on TAMs. We evaluated TAM targeting strategies using two novel MADs differing in molecular weight in a syngeneic mouse tumor model to determine how varying MAD molecular weights would impact tumor localization. Increased mass dose of the non-labeled construct or a higher molecular weight (HMW) construct were also used to block liver localization and enhance tumor to liver ratios.

**Procedures:**

Two MADs, 8.7 kDa and 22.6 kDa modified with DOTA chelators, were synthesized and radiolabeled with ^68^Ga. A HMW MAD (300 kDa) was also synthesized as a competitive blocking agent for Kupffer cell localization. Balb/c mice, with and without CT26 tumors, underwent dynamic PET imaging for 90 min followed by biodistribution analyses in selected tissues.

**Results:**

The new constructs were readily synthesized and labeled with ^68^Ga with ≥ 95% radiochemical purity in 15 min at 65 °C. When injected at doses of 0.57 nmol, the 8.7 kDa MAD provided 7-fold higher ^68^Ga tumor uptake compared to the 22.6 kDa MAD (2.87 ± 0.73%ID/g vs. 0.41 ± 0.02%ID/g). Studies with increased mass of unlabeled competitors showed reduced liver localization of the [^68^Ga]MAD-8.7 to varying degrees without significant reductions in tumor localization, resulting in enhanced tumor to liver signal ratios.

**Conclusion:**

Novel [^68^Ga]Manocept constructs were synthesized and studied in *in vivo* applications, showing that the smaller MAD localized to CT26 tumors more effectively than the larger MAD and that the unlabeled HMW construct could selectively block liver binding of [^68^Ga]MAD-8.7 without diminishing the localization to tumors. Promising results using the [^68^Ga]MAD-8.7 show a potential path to clinical applications.

**Graphical Abstract:**

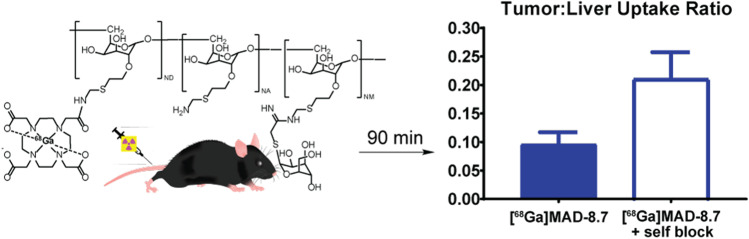

**Supplementary Information:**

The online version contains supplementary material available at 10.1007/s11307-023-01809-6.

## Introduction

Tumor associated macrophages (TAMs) are the most numerous immune cells in tumor microenvironments and important regulators of the immune status within tumors [1]. There are significant unmet clinical needs to image TAMs for either diagnostic or prognostic indications [2] and to target TAMs for cancer immunotherapies [3]. A significant proportion of TAMs and perhaps especially those TAMs that promote tumor progression express high levels of the macrophage mannose receptor (CD206) [4].

Navidea Biopharmaceuticals is developing a platform technology — the Manocept™ platform — of related molecular constructs with high binding affinity and specificity for CD206 [5]. Manocept-based compounds are built upon a dextran backbone to which mannose and various therapeutic and radiodiagnostic agents are appended via linkers. The multiple mannose sugars on Manocept constructs enable high affinity, multivalent binding to CD206 [6]. Manocept derivatives can be constructed with varied but defined molecular weight (Mw) profiles by selection of the appropriate Mw dextran at the initiation of synthesis. We hypothesized that smaller Mw constructs would diffuse into target tissues more effectively and provide greater localization versus those with a larger Mw.

Besides TAMs, CD206 is expressed by mesangial cells of the kidney and most classes of macrophages, including Kupffer cells of the liver [7, 8]*.* Kupffer cells are directly exposed to the blood flow, are highly numerous, and should quickly clear any CD206 ligands, including these proposed compounds, from the blood by CD206-mediated endocytosis [7, 9–11]. We hypothesized that Manocept constructs of relatively high Mw (HMW) would freely bind to CD206 on Kupffer cells but would be restricted from penetrating into tissues and binding to CD206 + on macrophages due to their larger sizes. Thus, intravenous injection (IV) of an excess of a HMW Manocept should competitively and selectively block binding of a low Mw Manocept to Kupffer cells of the liver while having minimal effect on the ability of a low Mw construct to penetrate tissues and bind to CD206 on macrophages.

This work reports results of imaging studies evaluating Manocept derivatives of varying Mw and labeled with ^68^Ga, with or without competitive Kupffer cell blocking in the CT26-Balb/c syngeneic murine colon cancer model.

## Materials and Methods

### Synthesis of Manocept™ Constructs

The chemical synthesis and characterization of the Manocept constructs, herein described as mannosylated amino dextrans (MADs), starting from 3.5 kDa dextran (final Mw = 8.7 kDa, MAD-8.7), 10 kDa dextran (final Mw = 22.6 kDa, MAD-22.6), and a HMW blocking agent on a 150 kDa dextran backbone (final Mw = 300 kDa, MAD-300 or HMW-MAD) are provided in the supplemental information and in US patent application #20210145989.

### Radiolabeling of [^68^Ga]-Manocept

To 0.57 nmol of desired precursor in 5 µL of 0.2 M ammonium acetate buffer, pH 5, was added ^68^GaCl_3_ (Eckert & Zeigler) in 98% acetone/0.01 M hydrochloric acid and up to an additional 10 µL of buffer. The reactions were incubated at 65 °C with agitation for 15 min, whereupon the radiochemical purity was assessed via instant thin layer chromatography (iTLC-SG, running buffer 0.5 M citric acid, pH 5). Prior to injection, final doses were diluted to 100 µL using PBS, pH 7.

### Radiometric CD206 Plate Binding Assay

Recombinant mouse MMR/CD206 protein (R&D Systems) was diluted to 10 µg/mL in coating buffer (15 mM Na_2_CO_3_/35 mM NaHCO_3_, pH 9.6). Approximately 48 h before the study, 100 µL (or 1 µg/well) of the protein solution was added to the wells and chilled at 2–8 °C. Day of study, compounds were radiolabeled as described previously then diluted to a final concentration of 100 nM in PBS. To all lanes, 100 µL of desired tracer was added. To bring the volume to 200 µL, non-blocking lanes had an additional 100 µL of PBS added and blocking lanes had 100 µL of 100-fold molar excess of unlabeled MAD-8.7 or MAD-22.6 or MAD-300 diluted in PBS. The plate was incubated at 37 °C for 1 h, whereupon the solution was removed, and the wells washed with 2 × 200 µL PBS. Individual wells and standards were assessed for radioactivity in a Hidex gamma counter, and data were decay corrected and expressed as a percent total activity.

### Animal Model

All animal studies were conducted in compliance with the guidelines for the care and use of research animals established by the University of Alabama at Birmingham’s Institutional Animal Care and Use Committee. CT26 cells were cultured according to ATCC product sheet in RPMI-1640 media containing 10% FBS and 0.1% Gentamicin at 37 °C with 5% CO_2_. Cells were prepared at a concentration of 2 × 10^6^ cells/100 µL in a 1:1 mix of PBS and Matrigel. BALB/c mice (Charles River) were anesthetized with 2–3% isoflurane in oxygen, and cells were subcutaneously implanted in the shoulder. Tumors were allowed to grow until palpable (≤ 1 cm^3^).

### PET Imaging Studies and Analysis

Day of study, mice were anesthetized with 2–3% isoflurane in oxygen and injected with ~ 3.7 MBq (100 µCi) of desired imaging tracer (0.57 nmol). For blocking studies, mice were given an injection of the selected blocking agent (250 µg in 25 µL of 0.1 M bicarbonate/carbonate buffer, pH 8) 5 min prior to tracer injection. Animals were kept sedated and maintained at a normal body temperature during imaging collection. The study consisted of 90 min of dynamic PET acquisition (energy window 350–650 keV) followed by a 5 min CT (80 kVp, 150 µA, 720 projections). The images were acquired with a Sofie GNEXT PET/CT scanner, and images were reconstructed using a 3D-OSEM (Ordered Subset Expectation Maximization) algorithm (24 subsets and 3 iterations), with random, attenuation, and decay correction. The CT images were reconstructed using a Modified Feldkamp Algorithm. Following image reconstruction, regions of interest (ROIs) were drawn for select tissues (the kidneys, livers, muscle, tumors) in each mouse using the CT, and time-activity curves (TAC) of the uptake of the tracer were generated over the course of the data collection. All image processing was performed using VivoQuant (Invicro).

### Biodistribution of Tracers in Select Tissues

At the conclusion of the image collection (~ 90 min post injection of the tracer), animals were euthanized, and tissues and organs of interest were collected. The radioactivity and weight of tissues were measured using an automated gamma counter (Hidex), and data were decay corrected to time of sacrifice and calculated as the percent injected dose per gram of tissue (%ID/g).

### Statistics

All statistical comparisons were performed using GraphPad Prism v9 and ordinary one-way ANOVA with Tukey’s multiple comparison tests. All cohorts were conducted with 4–6 mice (specified) and data were expressed as mean ± standard deviation.

## Results

### Synthesis of Manocept Constructs

MAD-8.7, MAD-22.6, and MAD-300 were prepared starting from commercially available 3.5 kDa, 10 kDa, and 150 kDa Mw dextran. Synthesis was initiated by appending amine terminated linkers via ether formation to the dextran backbone. A proportion of the amines were derivatized by covalent attachment of the metal chelator, 1,4,7,10-Tetraazacyclododecane-1,4,7,10-tetraacetic acid (DOTA), ~ 2 on the 3.5 kDa dextran backbone, and ~ 7 for the 10 kDa dextran backbone to enable efficient radiolabeling with gallium-68 (^68^Ga). A proportion of the remaining amines were further modified with mannose. Ten and 21 mannoses were added to the 3.5 kDa and 10 kDa backbones, respectively, providing MAD constructs with calculated Mw of 8.7 kDa (MAD-8.7) and 22.6 kDa (MAD-22.6) (Supplemental Fig. S1, compound 7a). The HMW blocking agent was synthesized on a 150 kDa dextran from which low Mw dextrans had been removed using a centrifuge spin-filter with a 100-kDa cutoff. Amine linkers and mannose were added at the same density as the smaller Mw constructs but did not receive a chelator moiety. The calculated Mw was ~ 300 kDa (MAD-300).

### Radiolabeling of ^68^Ga-MAD

[^68^Ga]MAD-8.7 and [^68^Ga]MAD-22.6 were successfully labeled with average radiochemical purities of 97.3 ± 1.9% (*n* = 6) and 98.9 ± 0.9% (*n* = 4), respectively. Labeling conditions were readily scaled for appropriate molar dose (0.57 nmol/animal) as well as radioactivity, up to 4 animal doses being prepared from the same batch. Representative iTLC-SG chromatograms are provided in Supplemental Fig. S2.

### Radiometric CD206 Plate Binding Assay

**Fig. 1:**
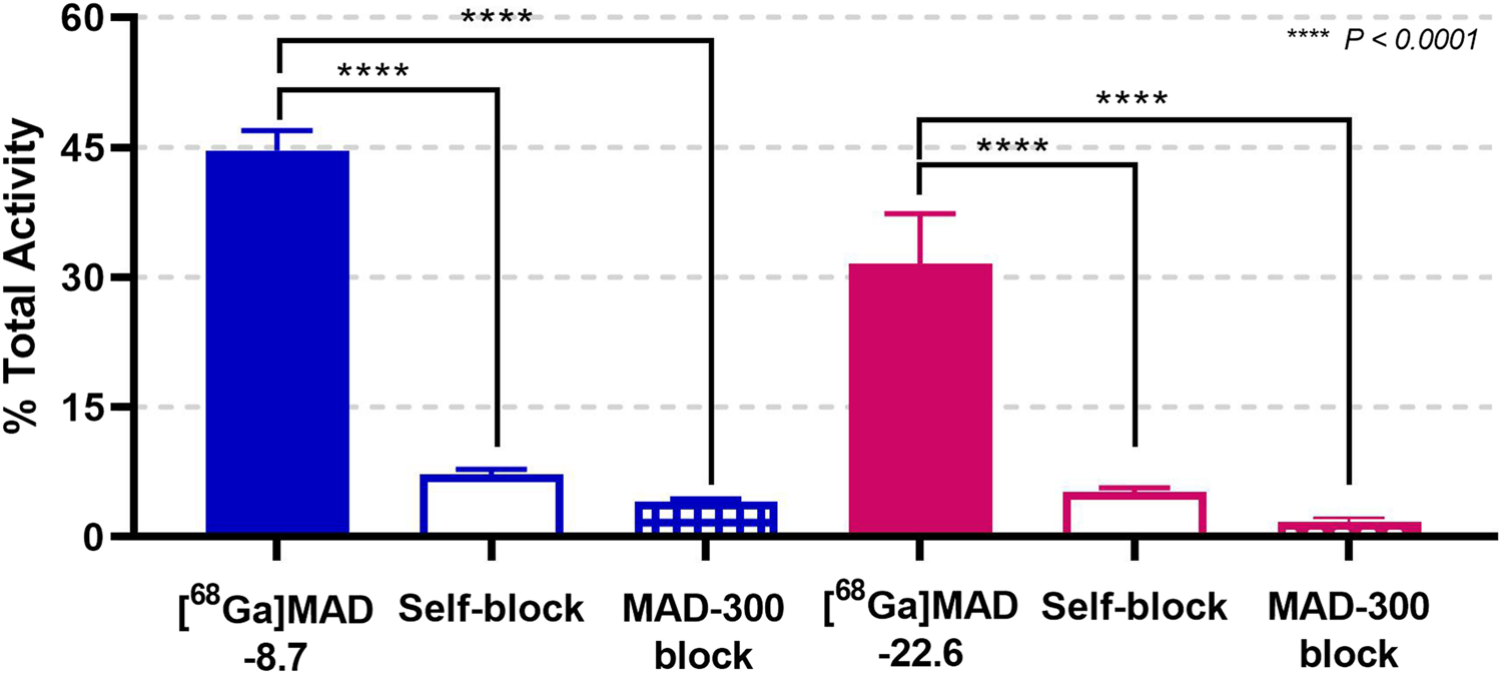
Radiometric plate binding assay of [^68^Ga]MAD-8.7 (blue) and [^68^Ga]MAD-22.6 to adhered CD206 under non-blocking and blocking conditions

The results from the plate binding assay can be seen in Fig. 1. Approximately, 45% of [^68^Ga]MAD-8.7 and 30% of [^68^Ga]MAD-22.6 bound to the CD206 under non-blocking conditions. When either radiotracer was incubated with 100-fold molar excess of its unlabeled construct or the HMW agent, MAD-300, binding of the tracers to the adhered CD206 decreased significantly (*p* < 0.0001*),* no matter the blocking agent used.

### PET Imaging Studies and Analysis

**Fig. 2:**
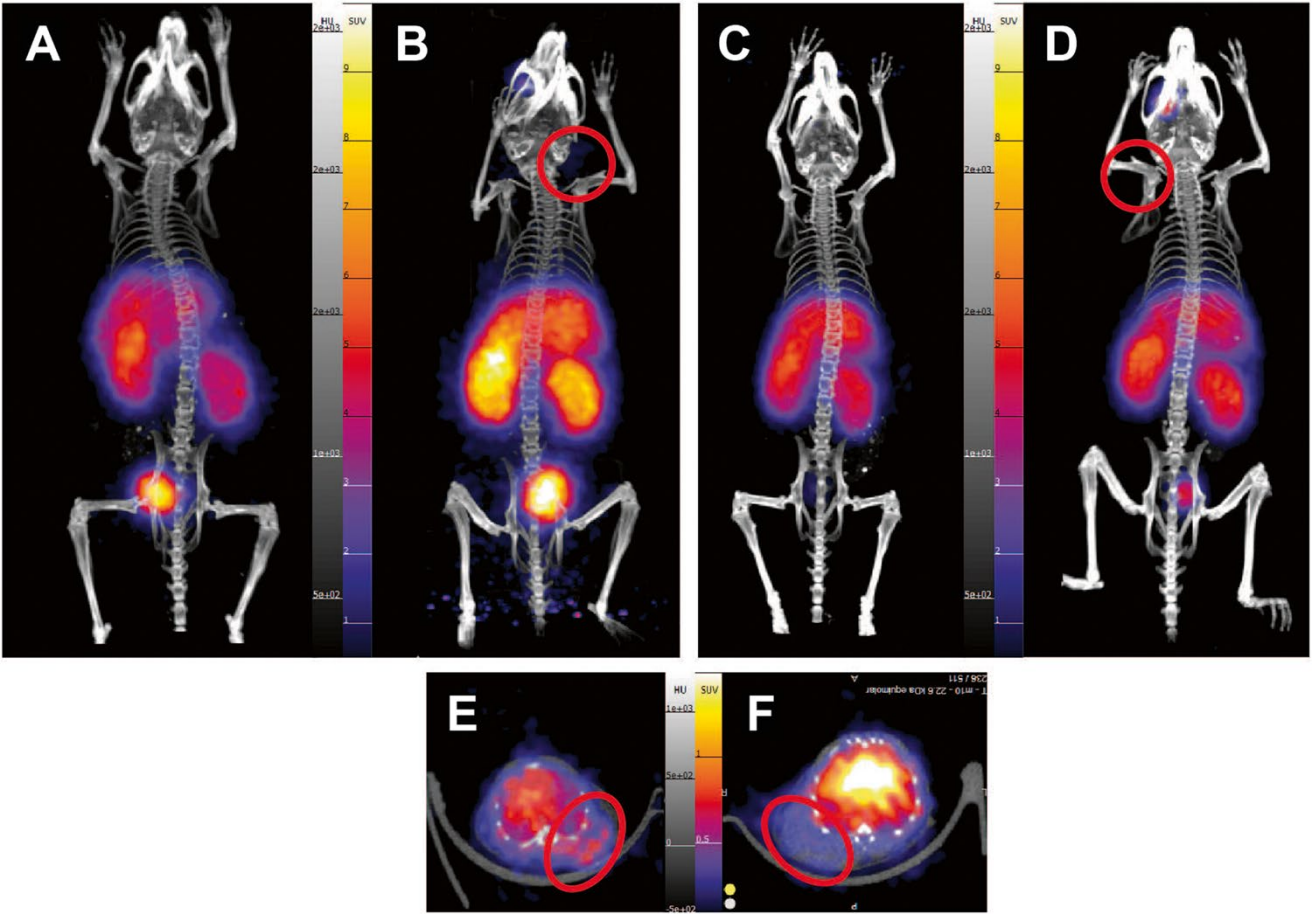
Representative maximal intensity projections (summed 80–90 min) illustrating uptake of [^68^Ga]MAD-8.7 in a control mouse (**A**) and a tumor bearing mouse (**B**) and uptake of [^68^Ga]MAD-22.6 in a control mouse (**C**) and a tumor bearing mouse (**D**). Circles are relative locations of tumors. Axial views illustrating liver and tumor localization for [^68^Ga]MAD-8.7 (**E**) and [^68^Ga]MAD-22.6, respectively. Circles in (**E**) and (**F**) are locations of tumors. The livers, kidneys and bladder are areas of high uptake and clearance

**Fig. 3:**
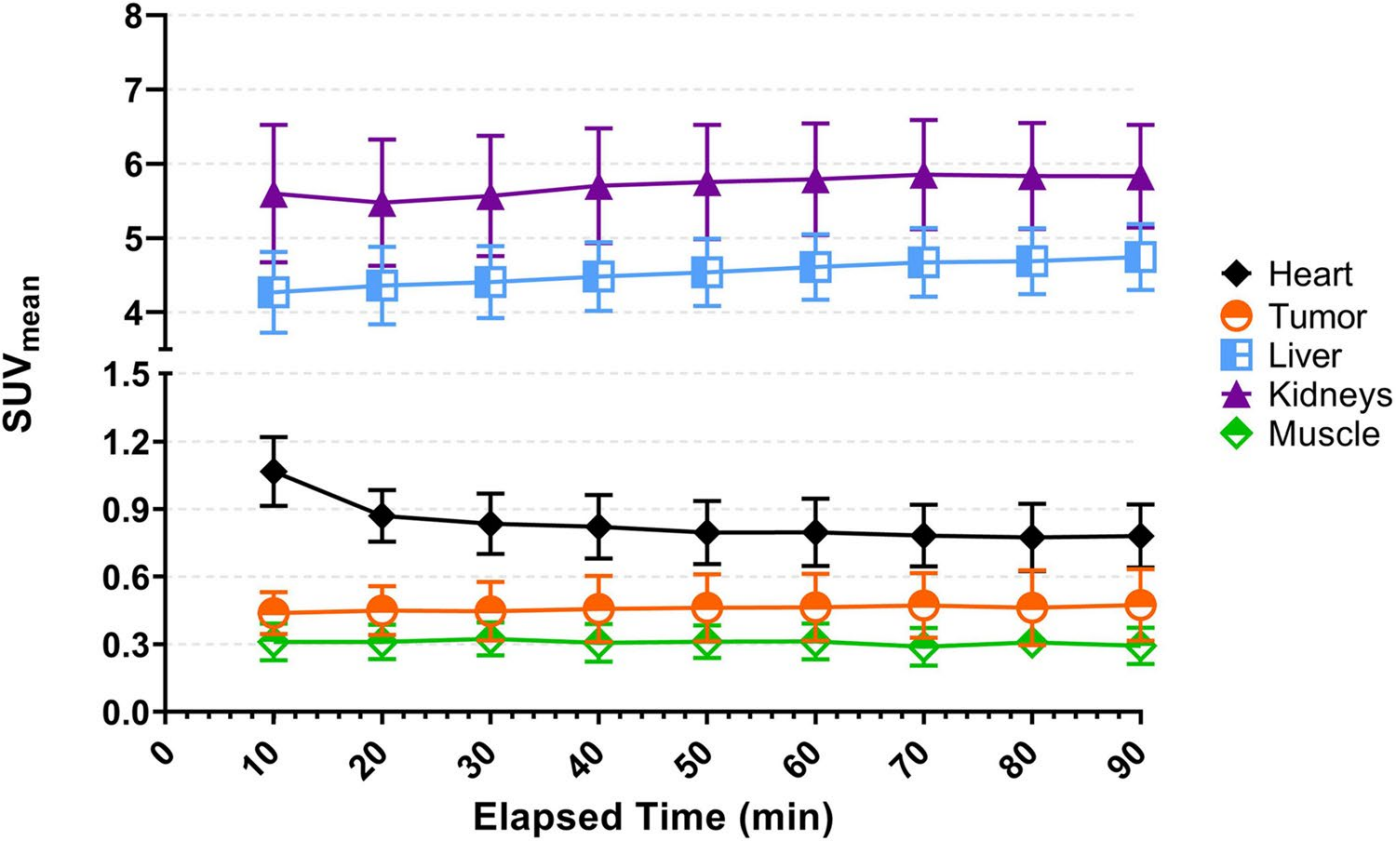
Time activity curve data for [^68^Ga]MAD-8.7 in tumor bearing mice. Data is presented as SUVmean over time in selected tissues. Error bars indicate standard deviation. *N* = 4 per tissue. Representative TAC data for [^68^Ga]MAD-22.6 for tumor bearing mice is presented Supplemental Fig. S4. TAC illustrate that tracers are in selected tissues within 10 min after injection, and levels stay relatively flat throughout the duration of the study

Representative maximal intensity projections (MIPs) and axial views of [^68^Ga]MAD-8.7 and [^68^Ga]MAD-22.6 in control and tumor bearing mice are seen in Fig. 2. Conspicuous localization of the imaging agents is noted in the kidneys, spleen, and liver where large numbers of CD206 + mesangial cells, macrophages, and Kupffer cells are known to reside. Bladder localization is likely due to urine excretion of the agents rather than localization to CD206 + cells. For comparison, distribution of the FDA approved Tc99m tilmanocept (Lymphoseek), a MAD construct with a 10-kD dextran backbone carrying a DTPA chelator, is shown in the Supplemental Fig. S3. A time activity curve (TAC) for [^68^Ga]MAD-8.7 localization in tumor bearing mice is illustrated in Fig. 3. This curve shows tissue uptake within 10 min after injection and that once the imaging agent localized in tissues (i.e., the kidneys, liver, muscle, or tumor), it did not migrate further. This result was observed in all groups, condition, and tissues tested. An additional TAC graph illustrating the trend for [^68^Ga]MAD-22.6 is illustrated in Supplemental Fig. S4.

### Biodistribution of Tracers in Select Tissues

**Fig. 4:**
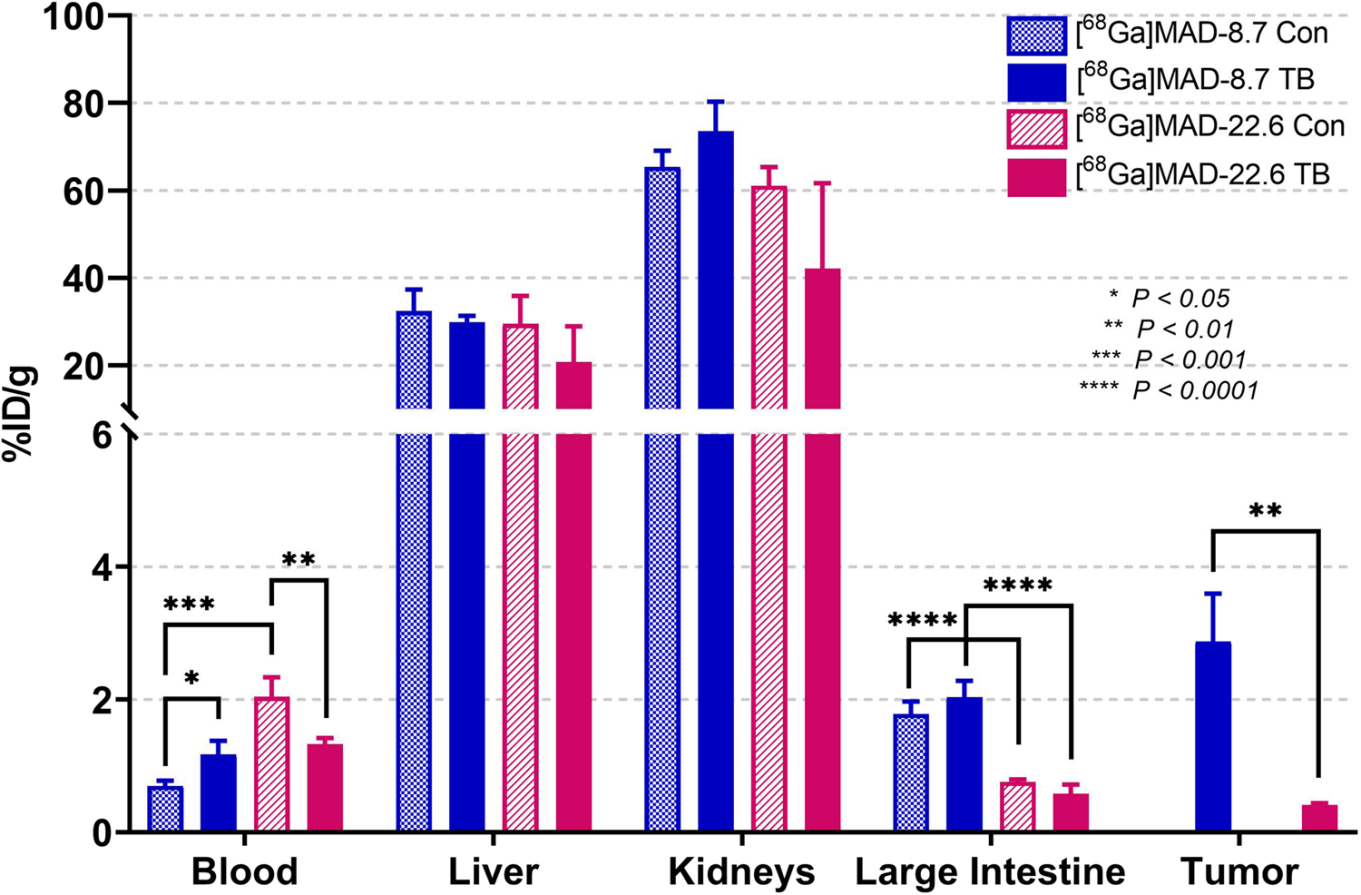
Post PET biodistribution results in select tissues at 90 min post injection. Error bars are indicative of standard deviation. *N* = 5 per control (Con) group and 4 per tumor bearing (TB) group. Complete biodistribution results are presented in table format in the supporting information, Supplemental Tables S1 and S2

Biodistribution results following injections of 0.57 nmol of [^68^Ga]MAD-8.7 and [^68^Ga]MAD-22.6 for selected tissues are illustrated in Fig. 4. The average size of tumors in tumor bearing (TB) mice was 0.45 g ± 0.30 g. There were few instances where biodistributions were significantly different between control and tumor bearing mice groups. One exception was residual activity in blood when using [^68^Ga]MAD-8.7 where control mice had 40% of the residual activity vs. the tumor bearing mice. However, the more significant difference observed was when comparing blood levels of [^68^Ga]MAD-8.7 to [^68^Ga]MAD-22.6. Overall, there was 3–6 times more [^68^Ga]MAD-22.6 found in the blood vs [^68^Ga]MAD-8.7 in either set of mice at 90 min post injection. Other important differences were observed in the [^68^Ga] uptake in the large intestine and the tumors. The smaller [^68^Ga]MAD-8.7 had over 2.4–3.5 times higher uptake (*p* < 0.0001) in large intestines vs. [^68^Ga]MAD-22.6. In tumors, [^68^Ga]MAD-8.7 had > 7-fold increased uptake vs. [^68^Ga]MAD-22.6: 2.87 ± 0.73%ID/g vs. 0.41 ± 0.02%ID/g, respectively. There was no significant difference in uptake of either imaging agent in the livers and kidneys of control or tumor bearing mice.

**Fig. 5:**
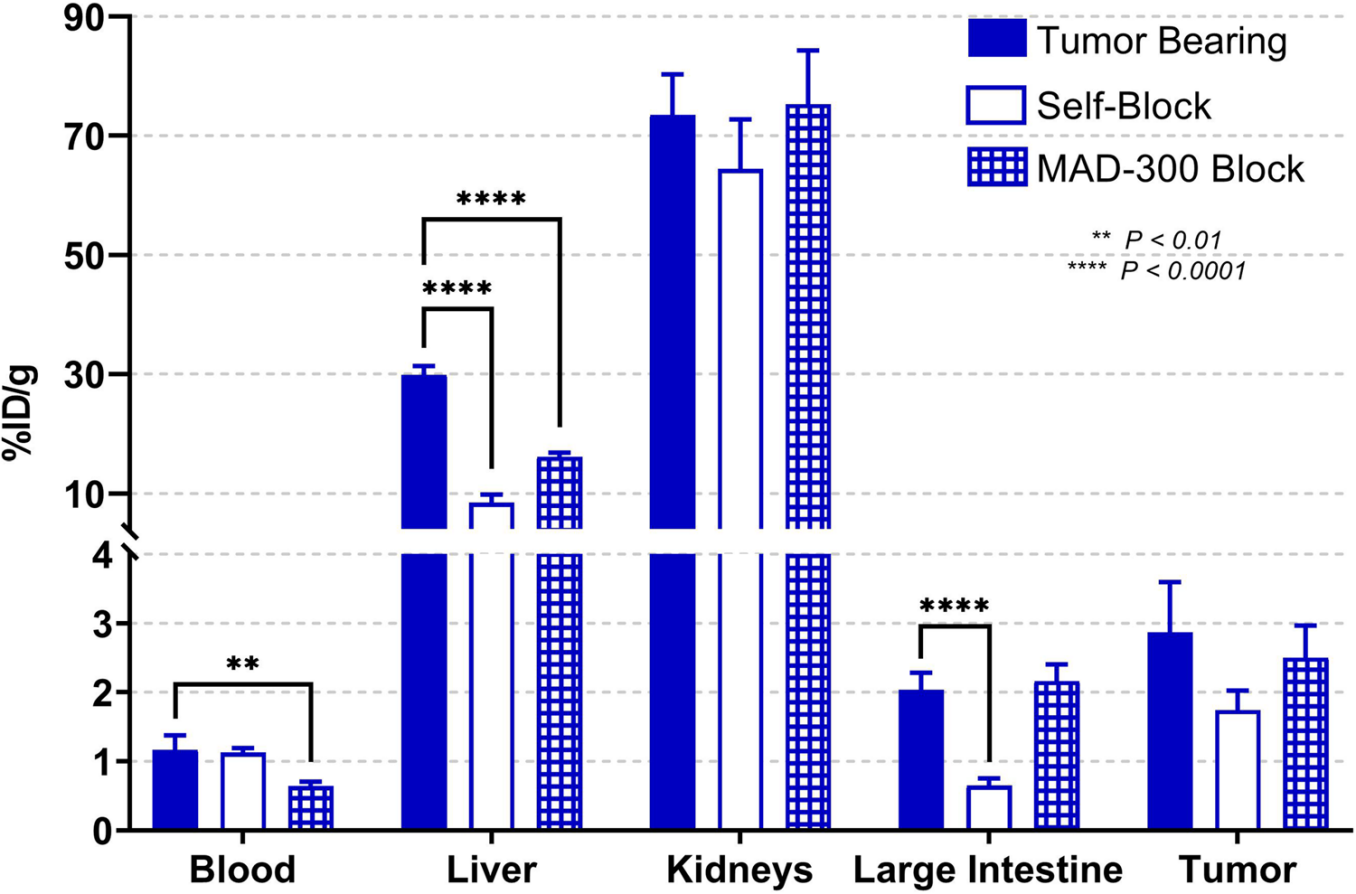
Biodistribution comparison of the tracer, [^68^Ga]MAD-8.7 under non-blocking and blocking conditions. Error bars indicate standard deviation. *N* = 4 per group. Remainder of biodistribution results are presented in table format in the supporting information, Supplemental Tables S1

**Fig. 6:**
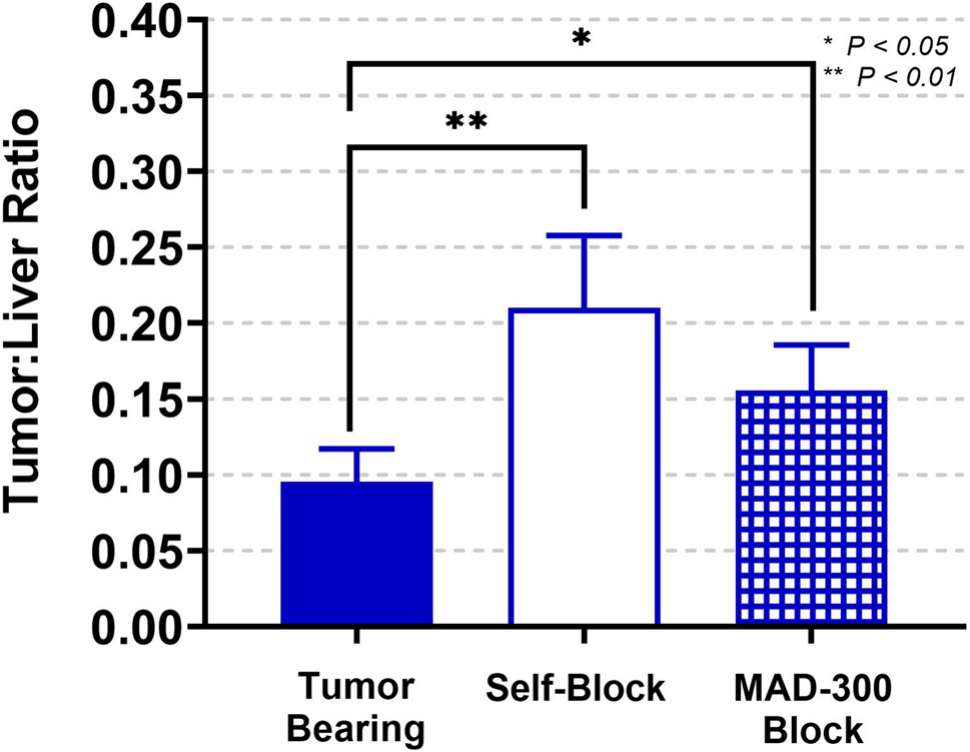
Tumor to liver uptake ratios of [^68^Ga]MAD-8.7 under non-blocking and blocking conditions

Figure 5 shows differences of [^68^Ga]MAD-8.7 uptake in various tissues and organs of tumor bearing mice that were injected with a 50-fold molar excess of unlabeled MAD-8.7 or 250 µg of MAD-300. Blood levels were lower when using the HMW compound, 0.64 ± 0.07%ID/g vs 1.16 ± 0.21%ID/g when self-blocking. Overall the uptake in tumors was not significantly affected when mice were pre-injected with unlabeled MAD constructs (i.e., blocking agents). In the large intestines, self-blocking significantly reduced [^68^Ga]MAD-8.7 localization by 68% (*p* < 0.0001), while blocking with HMW-MAD did not significantly alter localization. The liver was the only tissue measured that was significantly blocked independent of the molecular weight of the imaging agent or blocking compound used. Self-blocking and HMW-MAD blocking both significantly reduced (*p* < 0.0001 for both) liver localization of [^68^Ga]MAD-8.7 by 72% and 46%, respectively. This ultimately resulted in significantly increased tumor to liver uptake ratios when either self-blocking or blocking with HWM-MAD was used, Fig. 6.

## Discussion

Two MAD-based DOTA-carrying constructs were synthesized and demonstrated to bind specifically to CD206. The CD206 ligands were structurally similar, differing only in their molecular weights, 8.7 kDa and 22.6 kDa, respectively. Following labeling with [^68^Ga] and IV injection into Balb/c mice with and without CT26 tumors, biodistributions and TACs were generated. An *in vitro* plate assay where 50 nmol of [^68^Ga]MAD-8.7 or − 22.6 illustrated specific binding to surface-adhered CD206 and further illustrated the specificity by blocking with a 100-fold molar (5 µmol) excess of the labeling construct or the HMW agent, MAD-300. Representative MIPs (Fig. 2) show that the predominate sites of localization for either imaging agent in both control and tumor bearing mice were the livers and kidneys, known sites with large numbers of CD206 + Kupffer cells and mesangial cells, respectively. The liver, due to its relatively large size, localizes more imaging agent than other organs. These results are consistent with results obtained from mice injected with commercial Tc99m tilmanocept (Supplemental Fig. S3). Additionally, Fig. 2E–F shows axial views, illustrating tracer localization in tumors for both tracers.

In Fig. 3, the aggregate TAC data for the tumor bearing mice injected with the [^68^Ga]MAD-8.7 is representative of all time activity curves generated in these studies. Of note, beginning at the first measured time point taken ~ 10 min after injection, the mean SUV at all time points through 90-min generate time activity curves that approximate straight lines running parallel to the *x*-axis (time). This result has two implications. First, 10 min following injection, all or nearly all the injected dose of the imaging agent that could localize to a particular organ, has localized to that organ, further suggesting that the blood half-lives of these agents are much shorter than 10 min in mice. By comparison, Navidea has reported to the FDA that the blood half-lives of commercial Tc99m tilmanocept in rats and humans are approximately 15 min and 20 min, respectively. Second, once either the 8.7 kDa or 22.6 kDa imaging constructs have localized to an organ, they remain there stably and do not migrate to other sites. This is consistent with the known high affinities of similar MAD constructs to CD206 and their rapid internalization to endosomes [12].

One of the two primary goals of this study was to determine how the biodistribution of tilmanocept-like, DOTA derivatives would be altered by changing their molecular weights. Figure 4 shows biodistributions of the two tracers at the same molarity, in the blood, liver, kidneys, large intestine, and tumors observed after the 90-min imaging studies had been completed. In the livers and kidneys, there were no significant differences in the %ID/g localized to these organs for either MAD construct in either tumor bearing on non-bearing mice. This was as expected since there are no barriers or reduced barriers between the blood flow and the CD206 expressing cells (i.e., mesangial cells and Kupffer cells) in these organs. However, clear differences in localization of the imaging agents in the blood and in the large intestines and tumors were observed. The amounts of [^68^Ga]MAD-8.7 retained in the blood 90 min post injection were generally lower compared to [^68^Ga]MAD-22.6. In control mice without tumors, for [^68^Ga]MAD-8.7 and [^68^Ga]MAD-22.6, the %ID/g retained in the blood were 0.69 ± 0.08%ID/g and 2.04 ± 0.26%ID/g, respectively (*p* < 0.001). There were also non-significant differences in the amounts of the [^68^Ga]MAD constructs retained in the blood of tumor bearing mice. The reasons for these differences are currently not elucidated and will require further investigations to resolve but are probably related to other, non-CD206 mannose binding proteins found in blood, such as the mannose binding lectin [13], and to possible differences in binding affinities of different sized MAD constructs to these proteins.

It had been hypothesized that the smaller, 8.7 kDa construct would exit the blood flow and penetrate tissues, including tumors, more efficiently that the larger 22.6 kDa derivative. More efficient localization of smaller molecules relative to chemically similar, larger molecules is well established in the published literature [14, 15]. The results shown in Fig. 4 demonstrate that for both tumors and the large intestine, [^68^Ga]MAD-8.7 localized significantly more to these tissues compared to the larger 22.6 kDa construct. For the large intestine, 2.3 (non-tumor bearing, *p* < 0.0001) to 3.5 (tumor bearing, *p* < 0.0001) times more [^68^Ga]MAD-8.7 localized to the large intestines than [^68^Ga]MAD-22.6. In tumors, 7 times more [^68^Ga]MAD-8.7 localized to tumors than [^68^Ga]MAD-22.6 (*p* < 0.01). These results support the hypothesis that, by varying the sizes of MAD-based constructs, localization of ^68^Ga or other MAD payloads to tissues like tumors and the large intestine can be increased and optimized. These findings have important implications for designing MAD-based drug delivery vehicles targeting TAM directed cancer immunotherapies. The finding that [^68^Ga]MAD-8.7 localized more [^68^Ga]DOTA payload to the large intestines than [^68^Ga]MAD-22.6 may also have implications for designing MAD-based drug delivery vehicles targeting macrophages and dendritic cells involved in chronic inflammatory conditions of the large intestine or other organs [16–18].

Because of superior localizations of [^68^Ga]MAD-8.7 to large intestines and tumors, studies were performed to determine if competition with unlabeled MAD-8.7 or HMW-MAD could reduce liver localization without significantly reducing localizations to tumors and other potential target tissues as modeled by large intestines. In these studies, 250 μg of unlabeled MAD-8.7 or HMW-MAD were injected IV immediately prior to injection of 5 μg (0.57 nmol) of [^68^Ga]MAD-8.7. The resulting biodistributions of selected tissues are shown in Fig. 5. As was hypothesized, HMW-MAD significantly reduced the localization of [^68^Ga]MAD-8.7 to the liver by 46% (*p* < 0.0001) and did not significantly reduce localization to either the large intestines or tumors. These results have implications towards designing drug dosing protocols with a combination of a MAD-drug construct and an HMW-MAD for delivering small molecule therapeutics to TAMs and other sites of macrophage involved inflammation where off-target liver toxicity is a concern.

Also shown in Fig. 5 were results demonstrating that, similar to observations in the liver, competition with the HMW-MAD reduced retention of [^68^Ga]MAD-8.7 in the blood by 45% (*p* < 0.01). HMW-MAD also did not significantly alter the amount of [^68^Ga]MAD-8.7 that localized to the mesangial cells of the kidneys. This result was expected because HMW-MAD has an Mw of 300 kDa and is excluded from passing across glomeruli membranes due to its large size and, therefore, could not compete with [^68^Ga]MAD-8.7 for binding to CD206 on mesangial cells [19–21].

Contrary to the results returned with competitive blocking with the HMW-MAD, which were all consistent with expectations, competition with unlabeled MAD-8.7 (self-blocking) returned a series of unexpected results. It was expected that self-blocking would reduce the %ID/g retained in the blood similar to what was observed with HMW-MAD competition and that self-blocking would reduce localization of [^68^Ga]MAD-8.7 to the kidneys. The observed results were that self-blocking did not significantly alter the retention in blood or kidney localization of [^68^Ga]MAD-8.7. The observation in blood might indicate that [^68^Ga]MAD-8.7 is binding to an abundant entity in the blood with low affinity. It was also expected that self-blocking would reduce localization to the large intestines and tumors and to similar degrees. Instead, it was observed that self-blocking significantly reduced large intestine localization by 68% (*p* < 0.0001) but reduced tumor localization by a non-significant 39%. A full explanation of this observation will require further experimentation. However, self-blocking did, as expected, dramatically reduce liver localization of [^68^Ga]MAD-8.7 by 72% (*p* < 0.0001). This result might contribute to an explanation of why self-blocking did not reduce [^68^Ga]MAD-8.7 localization to the kidneys. Self-blocking, by dramatically reducing liver localization, may have permitted [^68^Ga]MAD-8.7 to stay in the blood circulation longer and allowing it greater opportunity to pass through and localize to the kidneys.

The results of these competitive blocking experiments permitted calculations of tumor to liver localization ratios (Fig. 6). Both self-blocking and HMW-MAD competition significantly increased tumor to liver ratios, but because self-blocking decreased liver localization to a greater extent than it reduced tumor localization, self-blocking increased the ratio more than did HMW-MAD competition. Combined with the results from large intestine localization, these results imply that for therapeutic indications for which liver toxicity is a concern, self-blocking of the liver may be preferred for TAM targeted cancer immunotherapies, while HMW-MAD competition may be preferred for indications related to treating inflammation at other sites such as the large intestine.

This report describes how the biodistributions of MAD-based diagnostic and therapeutic constructs may be beneficially altered by varying the molecular weights of both the MAD-based payload carrying constructs and unlabeled, payloadless MAD-based competitors. However, these results are limited in that neither the full molecular weight spectrum of MAD-payload and MAD-competitor constructs nor the full range of molar doses for the MAD-based constructs were explored. Furthermore, the reported studies revealed several unexpected results. Resolution of these issues will require additional investigations; however, with further investigation, it appears likely that MAD-based constructs and dosing protocols can be developed that optimize delivery of diagnostic imaging agents and/or therapeutic payloads to sites of macrophage involved inflammation while simultaneously minimizing delivery to off-target organs.

## Conclusion

In this report, two new ^68^Ga labeled, mannosylated amine dextran (MAD)–based, CD206 targeted imaging agents with different molecular weights were evaluated for their relative distributions in Balb/c mice with and without syngeneic CT26 tumors and with or without competitive self or HMW blocking. The four most important conclusions from this work were that (1) the imaging agents localized quickly to regions of interest in tissues and were retained stably for ≥ 90 min, (2) [^68^Ga]MAD-8.7 kDa localized 7-fold more to tumors than the [^68^Ga]MAD-22.6 kDa, (3) a HMW-MAD blocking agent can selectively and significantly reduce imaging agent localization to the liver without decreasing localization to potential target tissues such as tumors, and (4) competition with unlabeled MAD-8.7 kDa provided a higher tumor to liver localization ratio than HMW-MAD. These results have immediate implications for the rational design of mannosylated dextran-based imaging agents for tumors and other conditions in which large aggregations of CD206 + macrophages are known to occur. Furthermore, by replacing the chelator on the mannosylated dextran molecular construct with a therapeutic moiety, the results of these imaging studies inform the rational design of therapeutic constructs targeted to TAMs or macrophages associated with other conditions.

### Supplementary Information

Below is the link to the electronic supplementary material.

Supplementary file1 (DOCX 583 KB)

## Data Availability

The data that support the findings of this study are available from the corresponding author upon reasonable request.
