# Supplementary material for: In vivo Assessment of the Impact of Molecular Weight on Constructs of 68Ga-DOTA-Manocept in a Syngeneic Mouse Tumor Model
Source: Mol Imaging Biol. 2023 Mar 7;25(5):867–74. doi: 10.1007/s11307-023-01809-6 (PMC10598080; doi:10.1007/s11307-023-01809-6)
Supplement: Supplementary file 1 — Supplementary file1 (DOCX 583 KB) [file 11307_2023_1809_MOESM1_ESM.docx]

**Supplemental Information**

*In vivo* assessment of the impact of molecular weight on constructs of ^68^Ga-DOTA-Manocept in a syngeneic mouse tumor model

Jennifer L. Bartels,^1^ Solana R. Fernandez,^1^ Jeffrey Arnold^2^, Candace C. Parker^1^, Volkan Tekin^1^, Grace O’Malley^1^, David Ralph^2^, Suzanne E. Lapi^1*^

^1^Department of Radiology, The University of Alabama at Birmingham, Birmingham, AL 35294

^2^Navidea Biopharmaceuticals, 4995 Bradenton Ave, Dublin, Ohio, 43017

***Corresponding author.** Suzanne E. Lapi, 1824 6^th^ Ave. South, WTI 310F, Birmingham, AL, 35294, (p) 205-975-8689, (f) 205-934-0029, lapi@uab.edu

**Chemistry**

**Figure S1.**

**Figure S1**-Reagents and conditions: a. dextran, NaBH_4_, NaOH, C_3_H_5_Br, H_2_O, 70C, 18 h; b. allyl dextran **2**, (NH_4_)_2_S_2_O_8_, 2-aminoethanethiol, H_2_O, 55C 7 h; c. amine dextran **3**, DOTA-NHS, sodium carbonate pH 8.6; d. Cyanomethyl 2,3,4,5-tetra-O-acetyl-1-thio-a-D-mannopyranoside **5**, NaOMe, MeOH, rt 24-48 h;

e. Methyl 2-(a-D-mannopyranosylthio) ethanimidate **6**, sodium carbonate pH 8.6

**Allyl Dextran 2 on 10 kDa dextran**

A solution of 1.5 g of technical grade 10 kDa dextran (Pharmacosmos T-10) in 12 mL of distilled water was stirred at 35°C for 1 hour followed by the addition of 2.1 g (17.4 mmol, 116 equiv.) of allyl bromide, 0.75 g (18.7 mmol, 125 equiv.) of sodium hydroxide and 31.5 mg (0.83 mmol, 5.6 equiv.) of sodium borohydride in rapid succession. The contents of the flask were heated to 70°C while stirring vigorously under a water condenser. After 18 hours the reaction solution was cooled to ambient temperature, adjusted to pH 7 with acetic acid and transferred to an ultrafiltration stirred cell fitted with a 3 kDa MWCO membrane. The solution was concentrated and washed with distilled water, frozen and lyophilized providing allyl dextran **2** as a white foam (1.7 g). 1H NMR in deuterium oxide showed an average of 36 allyl groups per dextran chain (calc. Mw 11,441 g/mol).

**Amine Dextran 3 on 10 kDa dextran**

1.6 g of allyl dextran **2** was solubilized in distilled water at 0.15 g/mL and by heating and stirring at 50°C. 3.2 g (28.2 mmol) of 2-aminoethanethiol followed by 0.4 g (1.7 mmol) of ammonium persulfate were charged to the flask and the internal temperature increased to 55°C for 6 hours. The reaction solution was cooled to room temperature, neutralized with aqueous sodium hydroxide, and transferred to an ultrafiltration stirred cell with a 3 kDa MWCO membrane. The product was washed with distilled water to remove small molecular weight impurities and excess reagents, frozen and lyophilized. The process yielded 2.1 g of amino dextran **3** with complete conversion to 36 amine terminated linkers on the dextran backbone as determined by ^1^H NMR (calc. Mw 14,218 g/mol).

**Amine Dextran 3 on 3.5, and 150 kDa dextrans**

The same 2-step procedure was utilized to prepare allyl and amine dextrans **2** and **3** on 3.5 and 150 kDa technical grade dextrans with the exception that the 150 kDa material was washed with distilled water on a 100 kDa MWCO membrane to remove low Mw dextrans prior to derivatization. Processing resulted in an average of 17 amine chain linkers on the 3.5 kDa dextran and 531 on the 150 kDa dextran determined by ^1^H NMR analysis.

**DOTA 4 on 10 kDa Dextran**

150 mg of amine dextran **3** was solubilized in 0.1M sodium carbonate-bicarbonate pH 8.6 buffer at 25 mg/mL using brief sonication and stirring at ambient temperature for 30 minutes. A sample of the starting dextran solution was held as a time zero reference of reaction progress. Solid 1,4,7,10-Tetraazacyclododecane-1,4,7,10-tetraacetic acid (DOTA) activated as the mono *N*-hydroxysuccinimide ester was charged in portions to the amine dextran solution (40 mg then 25 mg) while following the conversion by monitoring the change in amine concentration compared to the starting solution via fluorescamine assay (CAS [38183-12-9], using a plate reader, Ex 390 nm, Em 475 nm). At the desired loading of DOTA, the reaction was concentrated with 0.1M aqueous sodium carbonate by 3 kDa MWCO ultrafiltration to remove free DOTA followed by concentration with purified water until the filtrate was a neutral pH. The retentate product solution was lyophilized providing 153 mg of DOTA dextran **4** as an off-white foam. The total DOTA loading was determined by spectrophotometric iron-chelation assay at 380 nm and was corrected for free (unbound) DOTA by HPLC analysis. The DOTA **4** product averaged 7 bound DOTA moieties per dextran chain and a calculated Mw of 17,382 g/mol.

**MAD-22.6 DOTA 7a**

126 mg of DOTA dextran **4** was solubilized in 0.1 M carbonate buffer pH 8.6 at 25 mg/ml and a sample retained as a T=0 sample for fluorescamine analysis. Methyl 2-(α-D-mannopyranosylthio) ethanimidate **6**, prepared via Zemplén deacetylation and imidate formation of **5**, was charged portion wise as a solid with intermittent fluorescamine analysis to monitor reaction progression. At the desired loading of mannose to the dextran backbone, the reaction solution was transferred to a stirred ultrafiltration cell fitted with a 3 kDa MWCO membrane, concentrated to minimal volume, and washed 4 times with distilled water. The retentate was filtered through a 0.45 µm syringe filter, frozen and lyophilized providing 122 mg of the MAD-22.6 DOTA construct as an off-white foam solid. By ^1^H NMR analysis, an average of 21 mannoses were bound to the dextran backbone making the final calculated Mw of the **7a** product 22.6 kDa. Conventional GPC analysis of this polymer using dextran standards, refractive index detection and aqueous conditions showed an Mw of 31.3 kDa, an Mn of 23.4 kDa and a low polydispersity index (PDI) value of 1.34, a PDI approximately equal to the dextran used at the start of synthesis. It should be noted that the GPC method utilizes dextrans for standard curve calibration, only providing dextran equivalent molar masses and not absolute values since the dextran has been modified.

**MAD-8.7 DOTA 7a**

The same 2-step procedure from amine dextran **3** on a 3.5 kDa dextran was utilized to prepare MAD-3.5 DOTA **7a**. The composition of the **7a** construct was an average of 1.9 DOTAs and 10 mannose sugars per dextran with a final calculated Mw of 8.7 kDa. Conventional GPC analysis of this polymer using dextran standards, refractive index detection and aqueous conditions showed an Mw of 14.4 kDa, an Mn of 10.8 kDa and a low polydispersity index (PDI) value of 1.33, a PDI approximately equal to the dextran used at the start of synthesis. It should be noted that this GPC method utilizes dextrans for standard curve calibration, only providing dextran equivalent molar masses and not absolute values since the dextran has been modified.

**MAD-300 Blocking Agent 8** (not shown in Figure S1)

500 mg of amine dextran **3** on the 150 kDa dextran scaffold was solubilized in 0.1 M carbonate buffer pH 8.6 at 25 mg/ml. Methyl 2-(α-D-mannopyranosylthio) ethanimidate **6**, prepared via Zemplén deacetylation and imidate formation of **5**, was charged portion wise as a solid with intermittent fluorescamine analysis to monitor reaction progression. At the desired loading of mannose to the dextran backbone, the reaction solution was transferred to a stirred ultrafiltration cell fitted with a 3 kDa MWCO membrane, concentrated to minimal volume, and washed 4 times with distilled water. The retentate was filtered through a 0.45 µm syringe filter, frozen and lyophilized providing 665 mg of MAD-300 **8** as an off-white foam solid. ^1^H NMR analysis showed an average of 342 mannoses bound to the dextran backbone making the final calculated Mw of the **8** product 293 kDa.

**Cy3-Labeled MAD-300 Blocking Agent** **and AF-488 Labeled MAD-8.7 for CD-206 Binding Affinity** (not shown in Figure S1)

Mannose amino dextrans of molecular mass 300 and 8.7 kDa were labeled with cyanine 3 and Alexa-Fluor 488 respectively by dissolving each MAD in 0.1 M carbonate buffer pH 8.6 followed by the addition of a DMSO solution of Cy3 or AF-488 activated as an *N*-hydroxysuccinimide ester. After 2 hours, the labeling reactions were transferred to centrifugal spin-filters (100 or 3 kDa MWCO), concentrated, and the retentates washed with water until the filtrates appeared colorless. The retentates were subsequently filtered through 0.2 um filters, frozen and lyophilized.

**Binding Assay**

Human peripheral blood monocytes were collected from a human donor. Monocytes were cultured for 5 days in RPMI-1640 medium supplemented with 10% FBS + 1X Penicillin/Streptomycin/L-glutamine + 50ng/ml GM-CSF. Following five (5) days of differentiation, GM-CSF-induced macrophages were harvested by cell scraping. Freshly thawed mononuclear cells (MNCs) were used as a negative control. Both macrophage and MNCs were stained with following markers as listed below in triplicate:

• Positive Control (CD206 [Clone 15-2] AlexaFluor488)

• Isotype Control (Mouse IgG1, κ Isotype Control AlexaFluor488)

• Negative Control (No Stain)

• Cy3/AF488 Labeled MAD Constructs: 1, 4, 16, 64 µg /ml

All cells were blocked with Human TruStain FcX prior to staining. Cells were stained with constructs and antibodies in PBS + 1mM MgCl2 for 15 minutes at 4°C. PI was used as the live/dead discriminator. All samples were acquired on a MACSQuant16 flow cytometer and analyzed in FlowJo. The portion of labelled cells and MFI of CD206 and CD206 Targeted Drug Delivery Vehicles for each sample were calculated. The experiment was performed in triplicate.

(A)
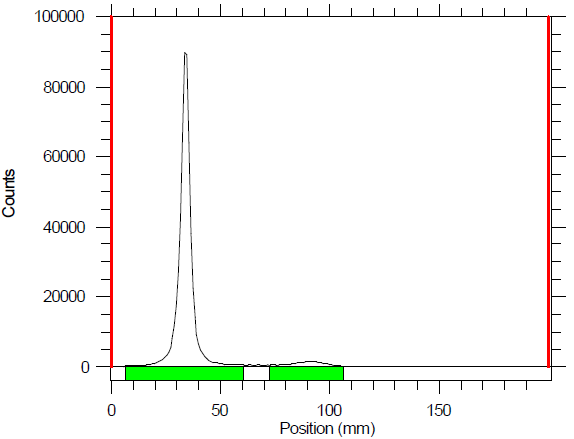
 (B)
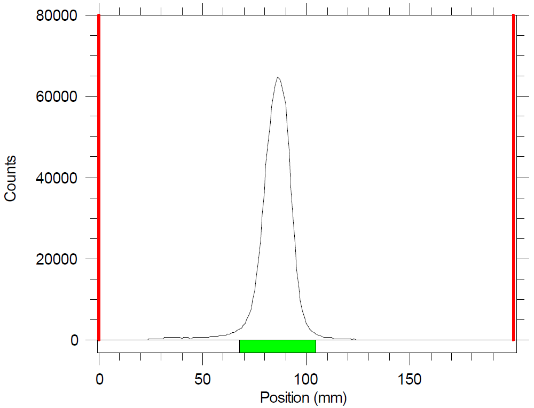


**Figure S2.** Radiometric iTLC’s of [^68^Ga]MAD-8.7 (A) vs free ^68^Ga (B) using the 0.5 M citric acid, pH 5 running buffer.


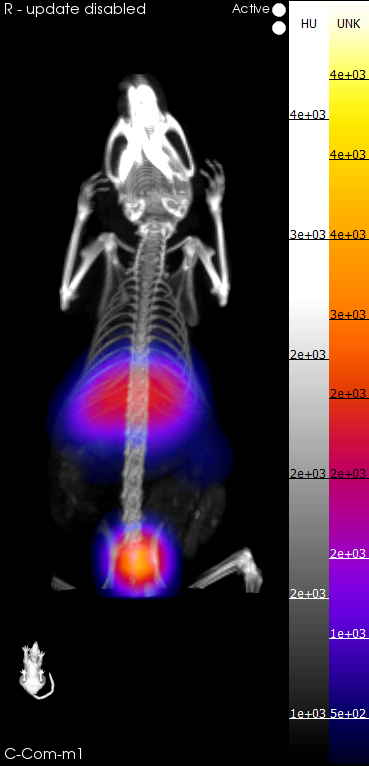


**Figure S3.**  Summed 80-90 min SPECT image of [^99m^Tc]Tilmanocept in a non-tumor bearing control mouse.


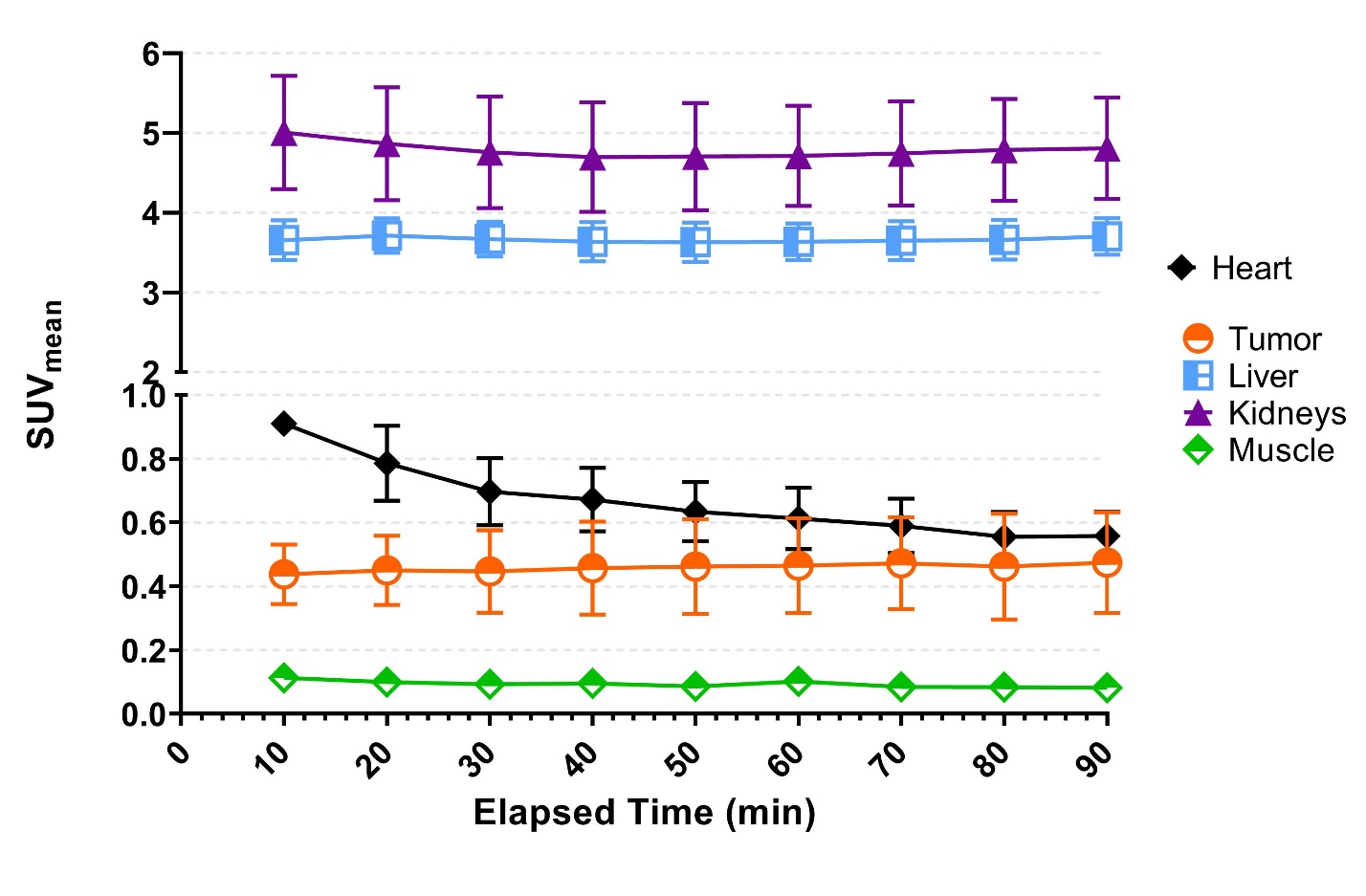


**Figure S4.** Time activity curve data for [^68^Ga]MAD-22.6 in tumor bearing mice. Data is presented as SUV_mean_ over time in selected tissues. Error bars indicate standard deviation. N = 4 per tissue.

|  | **Control** | | |  | | **Tumor Bearing** | | | |  | | **Self-Block** | | | |  | | **MAD-300 Block** | | | |  |
| --- | --- | --- | --- | --- | --- | --- | --- | --- | --- | --- | --- | --- | --- | --- | --- | --- | --- | --- | --- | --- | --- | --- |
|  | **Avg** |  | **StDev** | |  | | **Avg** |  | **StDev** | |  | | **Avg** |  | **StDev** | |  | | **Avg** |  | **StDev** | |
| **Blood** | 0.69 | +/- | 0.08 | |  | | 1.17 | +/- | 0.21 | |  | | 1.13 | +/- | 0.07 | |  | | 0.64 | +/- | 0.07 | |
| **Heart** | 3.30 | +/- | 0.20 | |  | | 3.21 | +/- | 0.20 | |  | | 1.12 | +/- | 0.15 | |  | | 3.33 | +/- | 0.67 | |
| **Lungs** | 2.23 | +/- | 0.18 | |  | | 2.52 | +/- | 0.10 | |  | | 1.64 | +/- | 0.21 | |  | | 2.91 | +/- | 0.43 | |
| **Spleen** | 16.27 | +/- | 6.52 | |  | | 11.74 | +/- | 2.07 | |  | | 9.10 | +/- | 1.17 | |  | | 9.16 | +/- | 1.82 | |
| **Liver** | 32.40 | +/- | 4.97 | |  | | 29.85 | +/- | 1.48 | |  | | 8.47 | +/- | 1.38 | |  | | 16.09 | +/- | 0.68 | |
| **Kidneys** | 65.44 | +/- | 3.64 | |  | | 73.51 | +/- | 6.78 | |  | | 64.42 | +/- | 8.30 | |  | | 75.29 | +/- | 9.00 | |
| **S. Intestines** | 1.69 | +/- | 0.44 | |  | | 2.06 | +/- | 0.23 | |  | | 0.79 | +/- | 0.29 | |  | | 2.04 | +/- | 0.10 | |
| **L. Intestines** | 1.78 | +/- | 0.19 | |  | | 2.03 | +/- | 0.25 | |  | | 0.65 | +/- | 0.11 | |  | | 2.16 | +/- | 0.24 | |
| **Skin** | 0.97 | +/- | 0.14 | |  | | 1.10 | +/- | 0.20 | |  | | 0.61 | +/- | 0.05 | |  | | 1.03 | +/- | 0.24 | |
| **Muscle** | 0.46 | +/- | 0.12 | |  | | 0.43 | +/- | 0.15 | |  | | 0.19 | +/- | 0.04 | |  | | 0.45 | +/- | 0.09 | |
| **Bone** | 2.05 | +/- | 0.55 | |  | | 2.47 | +/- | 0.35 | |  | | 1.38 | +/- | 0.52 | |  | | 1.57 | +/- | 0.49 | |
| **Brain** | 0.08 | +/- | 0.03 | |  | | 0.10 | +/- | 0.03 | |  | | 0.06 | +/- | 0.01 | |  | | 0.12 | +/- | 0.04 | |
| **Tumor** | -- | +/- | -- | |  | | 2.87 | +/- | 0.73 | |  | | 1.74 | +/- | 0.28 | |  | | 2.50 | +/- | 0.47 | |

**Table S1.** Biodistribution data (average %ID/g +/- standard deviation) of 0.57 nmol of [^68^Ga]MAD-8.7 injected in all tissues and control, tumor bearing and blocking conditions at ~90 min post injection.

|  | **Control** | | |  | **Tumor Bearing** | | |  |
| --- | --- | --- | --- | --- | --- | --- | --- | --- |
|  | **Avg** |  | **StDev** |  | **Avg** |  | **StDev** |  |
| **Blood** | 2.04 | +/- | 0.26 |  | 1.32 | +/- | 0.08 |  |
| **Heart** | 1.80 | +/- | 0.14 |  | 1.56 | +/- | 0.32 |  |
| **Lungs** | 1.64 | +/- | 0.29 |  | 1.25 | +/- | 0.35 |  |
| **Spleen** | 13.88 | +/- | 6.62 |  | 8.42 | +/- | 3.61 |  |
| **Liver** | 29.44 | +/- | 5.59 |  | 20.73 | +/- | 6.69 |  |
| **Kidneys** | 61.10 | +/- | 3.70 |  | 42.06 | +/- | 16.04 |  |
| **S. Intestines** | 1.58 | +/- | 0.17 |  | 1.18 | +/- | 0.23 |  |
| **L. Intestines** | 0.75 | +/- | 0.03 |  | 0.58 | +/- | 0.11 |  |
| **Skin** | 0.68 | +/- | 0.04 |  | 0.32 | +/- | 0.07 |  |
| **Muscle** | 0.15 | +/- | 0.03 |  | 0.14 | +/- | 0.03 |  |
| **Bone** | 1.93 | +/- | 0.77 |  | 0.95 | +/- | 0.13 |  |
| **Brain** | 0.12 | +/- | 0.06 |  | 0.21 | +/- | 0.09 |  |
| **Tumor** | -- | +/- | -- |  | 0.41 | +/- | 0.02 |  |

**Table S2.** Biodistribution data (average %ID/g +/- standard deviation) of 0.57 nmol of [^68^Ga]MAD-22.6 injected in all tissues in control and tumor bearing mice at ~90 min post injection.


| **CD206+ Macrophages (TAMs) in CT26 Tumors** | | |
| --- | --- | --- |
|  | % | StDev (%) |
| CD45+ Cells as % of Total Cells | 26.0 | 4.50 |
| TAMs as % of CD45+ Cells | 48.7 | 4.45 |
| TAMs as % Total Cells | 12.6 | 2.25 |
| CD206+ TAMs as % of All TAMs | 51.7 | 9.10 |
| CD206+ TAMs as % of Total Cells | 6.49 | 1.42 |
| Macrophages (TAMs) are CD45+, F4/80+, CD11b+ cells | | |

**Table S3.** Balb/c Mice were implanted with CT26 tumor cells. When the tumors reached 80-100 mm^3^, mice (n = 10), mice were treated 3 times with IV injected saline (treatment days 1, 4, and 7). Tumors were harvested (day 8) and disaggregated into a single cell suspension and evaluated by flow cytometry for the cellular composition of infiltrating immune cells. All Tumors < 1000 mm^3^ at collection. Results for TAMs are shown indicating the proportion of TAMs that were CD206+. An average of 373,000 cells +/- 150,000) per tumor were examined.

|  | **MFI-Macs** | **MFI-MNC** |
| --- | --- | --- |
| CD206 AF488 | 23560 | 28 |
| Isotype AF488 | 739 | 28 |
| Unstained | 144 | 5 |
| HMW-MAD Cy3 -1 µg/mL | 416 | 19 |
| HMW-MAD Cy3 -4 µg/mL | 1379 | 261 |
| HMW-MAD Cy3 -16 µg/mL | 6988 | 1812 |
| HMW-MAD Cy3 -64 µg/mL | 26869 | 2910 |

**Table S4.** Results of binding assay evaluating HMW-MAD-Cy3 localization to GM-CSF stimulated human macrophages (CD206+) and fresh human monocytes (MNC, CD206-) compared to Alexa Fluor 488 labelled anti-CD206 antibody and isotype antibody control, and unstained cells. Numerical values are average MFI from 3 replicate experiments. At concentrations above 1 µg/ml, nearly all macrophages had detectable localization of HMW-MAD-Cy.


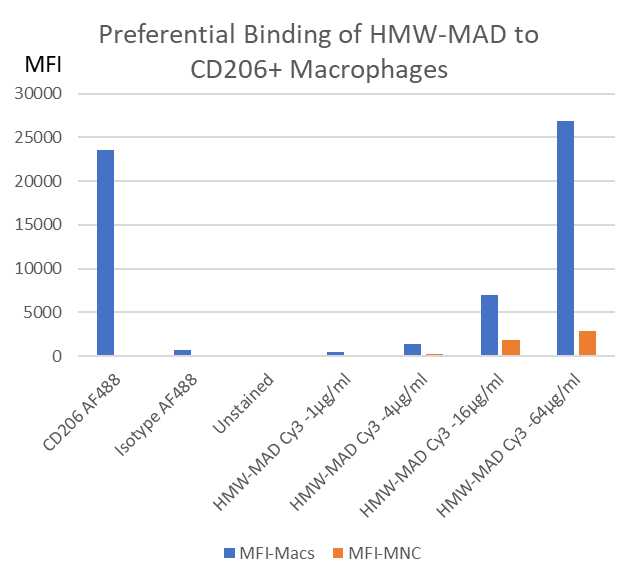


**Figure S5**. Graph of results shown in the previous Table demonstrating that HMW-MAD-Cy3 binds preferentially to CD206+ macrophages compared to CD206- MNC.

|  | **MFI-Macs** | **MFI-MNC** |
| --- | --- | --- |
| CD206 AF488 | 23053 | 36 |
| Isotype AF488 | 769 | 36 |
| Unstained | 706 | 39 |
| MAD Pre8.7 -1 µg/mL | 16623 | 38 |
| MAD Pre8.7 -4 µg/mL | 19516 | 38 |
| MAD Pre8.7 -16 µg/mL | 22404 | 39 |
| MAD Pre8.7 -64 µg/mL | 36316 | 57 |

**Table S5.** Results of binding assay evaluating the MAD-8.7 precursor (before adding DOTA and labeled with Alexa Fluor 488, MAD Pre8.7) localization to GM-CSF stimulated human macrophages (CD206+) and fresh human monocytes (MNC, CD206-) compared to Alexa Fluor 488 labelled anti-CD206 antibody and isotype antibody control, and unstained cells. Numerical values are average MFI from 3 replicate experiments. Nearly all macrophages had detectable localization of MAD Pre8.7 Alexa Fluor 488 at all concentrations evaluated.

MFI

**Figure S6.** Graph of results shown in the previous Table demonstrating that MAD-Pre8.7 Alexa Fluor 488 binds preferentially to CD206+ macrophages compared to CD206- MNC.
